# Temporal Trends in Suicide Methods Among Adolescents in the US

**DOI:** 10.1001/jamanetworkopen.2022.36049

**Published:** 2022-10-12

**Authors:** Victoria A. Joseph, Gonzalo Martínez-Alés, Mark Olfson, Jeffrey Shaman, Madelyn S. Gould, Katherine M. Keyes

**Affiliations:** 1 Department of Epidemiology, Mailman School of Public Health, Columbia University, New York, New York; 2 CAUSALab, Harvard University T. H. Chan School of Public Health, Boston, Massachusetts; 3 Mental Health Network Biomedical Research Center (CIBERSAM), Madrid, Spain; 4 Hospital La Paz Institute for Health Research, Madrid, Spain; 5 Department of Psychiatry, Columbia University, New York, New York; 6 Department of Environmental Health Sciences, Columbia University Mailman School of Public Health, Columbia University, New York, New York

## Abstract

This cross-sectional study evaluates the temporal trends in suicide methods among US adolescents, with variation by sex and race.

## Introduction

Deaths due to suicide increased 45.2% in the past 10 years among adolescents in the US,^1^ with disproportionate increases among youths who are members of minority groups.^2,3^ Method of suicide is a strong determinant of suicide fatality, and research on temporal trends in suicide methods among decedents is scarce, especially by race. To address concerns regarding increasing suicide rates, we examined temporal trends in suicide methods among adolescents, with attention to variation by sex and race.

## Methods

This cross-sectional time series followed the STROBE reporting guideline. Owing to the use of publicly available data from the Centers for Disease Control and Prevention WONDER database, institutional review board approval and informed consent were not necessary according to the Common Rule.

Suicide death data for adolescents aged 10 to 19 years and stratified by sex and race were drawn from the National Center for Health Statistics’ Multiple Cause of Death files from 1999 to 2020.^1^ Racial data were collected owing to increasing suicide rates among members of minority groups. Racial categories included American Indian or Alaska Native, Asian or Pacific Islander, Black or African American, and White. Ethnicity data were not included in the present study. Method of suicide was categorized as firearm, asphyxiation (including hanging, strangulation, and suffocation), and a single category comprising other methods (poisoning, drowning, fall, fire, and cuts) due to lower base frequency. Locally estimated scatterplot smoothing regression curves with 95% CIs and logistic regression models were used to evaluate the interaction of time (5-year periods) on the association between race and suicide method. Statistical significance was set at *P* < .05.

## Results

From 1999 to 2020, 47 276 adolescents aged 10 to 19 years (3.0% American Indian or Alaska Native, 4.0% Asian or Pacific Islander, 11.0% Black or African American, and 82.0% White; 23.0% female and 77.0% male) died by suicide in the US. Suicide rates increased steadily for male adolescents, from 7.4 to 9.7 per 100 000 population; for female adolescents, from 1.6 to 3.6 per 100 000 population. Among male adolescents who died by suicide, firearms remained the leading suicide method (Figure), but trends differed substantially by race, with firearms increasingly accounting for deaths among racial minority youths. From 2011 to 2020, the proportion of suicide deaths involving firearms increased from 40.0% to 51.0% among Black male adolescents compared with 49.0% to 52.0% among White male adolescents (Table). Among female adolescents, asphyxiation was the leading method since 2000 (Figure). Suicide death by asphyxiation increased from 53.0% in 1999 to 2001 to 74.0% in 2017 to 2020 among American Indian or Alaska Native female adolescents compared with 37.0% to 52.0% among their White counterparts (Table).

**Figure.**
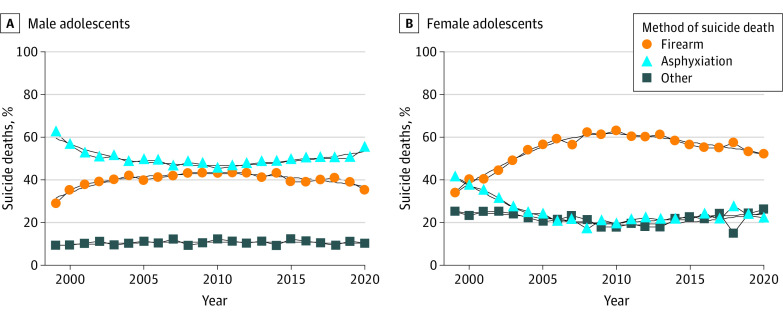
Percentage of Suicide Deaths by Method Among Male and Female Adolescents Aged 10 to 19 Years, 1999-2020 Includes observed percentages of suicide deaths by method among male and female adolescents with locally estimated scatterplot smoothing regression estimated percentages of suicide deaths by method with 95% CIs. Asphyxiation includes suicide deaths involving hanging, strangulation, and suffocation; firearm includes suicide deaths involving firearm use; and other includes suicide deaths involving poisoning, drowning, fall, fire, and cuts. Data are from the Centers for Disease Control and Prevention.^1^

**Table.** Percentages of Suicide Death by Sex, Method, and Race Among US Adolescents Aged 10 to 19 Years From 1999 to 2020

Year	Means of suicide by race, %											
	Firearm^a^				Asphyxiation^b^				Other^c^			
	American Indian or Alaska Native	Asian or Pacific Islander	Black or African American	White	American Indian or Alaska Native	Asian or Pacific Islander	Black or African American	White	American Indian or Alaska Native	Asian or Pacific Islander	Black or African American	White
**Female adolescents**												
1999-2001	28.0	20.0	36.0	39.0	53.0	48.0	40.0	37.0	19.0	32.0	24.0	24.0
2002-2004	15.0	16.0	29.0	28.0	73.0	55.0	51.0	48.0	12.0	29.0	20.0	24.0
2005-2007	15.0	9.0	20.0	23.0	75.0	63.0	59.0	55.0	10.0	28.0	21.0	22.0
2008-2010	8.0	8.0	11.0	22.0	79.0	67.0	77.0	59.0	13.0	25.0	12.0	81.0
2011-2013	9.0	11.0	12.	24.0	89.0	52.0	75.0	57.0	2.0	37.0	13.0	19.0
2014-2016	6.0	13.0	18.0	24.0	85.0	54.0	56.0	55.0	9.0	33.0	26.0	21.0
2017-2020	12.0	15.0	21.0	24.0	74.0	58.0	58.0	52.0	14.0	27.0	21.0	24.0
**Male adolescents**												
1999-2001	46.0	39.0	62.0	57.0	52.0	45.0	31.0	33.0	2.0	16.0	7.0	10.0
2002-2004	42.0	34.0	50.0	50.0	51.0	44.0	43.0	39.0	7.0	22.0	7.0	11.0
2005-2007	40.0	29.0	53.0	48.0	48.0	52.0	39.0	41.0	12.0	19.0	8.0	11.0
2008-2010	34.0	22.0	45.0	48.0	58.0	59.0	46.0	41.0	8.0	19.0	9.0	11.0
2011-2013	36.0	28.0	40.0	49.0	60.0	54.0	50.0	40.0	4.0	18.0	10.0	11.0
2014-2016	44.0	28.0	45.0	51.0	51.0	52.0	42.0	39.0	5.0	20.0	13.0	10.0
2017-2020	43.0	32.0	51.0	52.0	51.0	45.0	39.0	38.0	6.0	23.0	10.0	10.0

^a^ Includes all suicide deaths involving firearm use.

^b^ Includes suicide deaths involving hanging, strangulation, and suffocation.

^c^ Includes suicide deaths involving poisoning, drowning, fall, fire, and cuts.

Logistic regression models evaluating the association between race and death by asphyxiation vs other methods and between firearms vs other methods, stratified by year and sex, indicated statistically significant interactions. For instance, in 2019 to 2020, Black female adolescents had 1.43 (95% CI, 1.05-1.95) times the odds of suicide death involving asphyxiation vs all other methods compared with their White counterparts, whereas from 1999 to 2003 these odds were 1.06 (95% CI, 0.74-1.52).

## Discussion

This time series found that suicide deaths by asphyxiation increased over time among female adolescents who were members of minority groups, whereas firearms remained the predominant method of suicide death among male adolescents. Furthermore, the proportions of suicide deaths involving firearms among Black male adolescents increased at a much faster pace than that among other racial groups.

Prevention of suicide involving firearms through restriction of access remains urgent.^4^ The results of this study suggest an additional need to expand suicide prevention initiatives. Reducing access to asphyxiation means is difficult outside of institutionalized settings; thus, a focus on reducing the frequency and intensity of suicidal crises is critical. The emergence of suicide as a public health concern among Black or African American and Asian or Pacific Islander adolescents indicates a need for culturally adaptive, structurally competent approaches to ensure access to mental health services.

Limitations of this study include potential errors in suicide mortality certification and underreporting of suicide deaths, especially for members of minority groups.^5^ Future studies should consider assessing age, state-level differences, and trends of method of suicide deaths, including clinical characteristics and ethnicity of adolescents.^6^
