# Error in the Figure

**DOI:** 10.1001/jamanetworkopen.2022.43633

**Published:** 2022-10-28

In the Research Letter titled “Temporal Trends in Suicide Methods Among Adolescents in the US,”^1^ published on October 12, 2022, there was an error in the Figure. The symbols in the key for firearm deaths and asphyxiation deaths were inadvertently transposed. This article has been corrected.^1^
